# Prenatal exposure to organophosphate pesticides and risk-taking behaviors in early adulthood

**DOI:** 10.1186/s12940-021-00822-y

**Published:** 2022-01-10

**Authors:** Sharon K. Sagiv, Stephen Rauch, Katherine R. Kogut, Carly Hyland, Robert B. Gunier, Ana M. Mora, Asa Bradman, Julianna Deardorff, Brenda Eskenazi

**Affiliations:** 1 Center for Environmental Research and Community Health (CERCH), School of Public Health, University of California, 1995 University Avenue, Suite 265, Berkeley, CA 94720 USA; 2 Department of Public Health, University of California, Merced, CA USA

**Keywords:** Pesticides, Organophosphates, Prenatal exposure, Risky behavior, Substance use, Sexual behavior, Risky driving, Delinquency

## Abstract

**Introduction:**

Previous studies show evidence for associations of prenatal exposure to organophosphate (OP) pesticides with poorer childhood neurodevelopment. As children grow older, poorer cognition, executive function, and school performance can give rise to risk-taking behaviors, including substance abuse, delinquency, and violent acts. We investigated whether prenatal OP exposure was associated with these risk-taking behaviors in adolescence and young adulthood in a Mexican American cohort.

**Methods:**

We measured urinary dialkyl phosphates (DAPs), non-specific metabolites of OPs, twice (13 and 26 weeks gestation) in pregnant women recruited in 1999–2000 in the Center for the Health Assessment of Mothers and Children of Salinas (CHAMACOS) study, a birth cohort set in a primarily Latino agricultural community in the Salinas Valley, California. We followed up children throughout their childhood and adolescence; at the 18-year visit, adolescent youth (*n* = 315) completed a computer-based questionnaire which included questions about substance use, risky sexual activity, risky driving, and delinquency and police encounters. We used multivariable models to estimate associations of prenatal total DAPs with these risk-taking behaviors.

**Results:**

The prevalence of risk-taking behaviors in CHAMACOS youth ranged from 8.9% for smoking or vaping nicotine to 70.2% for committing a delinquent act. Associations of total prenatal DAPs (geometric mean = 132.4 nmol/L) with risk-taking behavior were generally null and imprecise. Isolated findings included a higher risk for smoking or vaping nicotine within the past 30 days (relative risk [RR] per 10-fold increase in prenatal DAPs = 1.89, 95% CI: 1.00, 3.56) and driving without a license (RR = 1.74, 95% CI: 1.25, 2.42). There were no consistent differences by sex or childhood adversity.

**Discussion:**

We did not find clear or consistent evidence for associations of prenatal OP exposure with risk-taking behaviors in adolescence/early adulthood in the CHAMACOS population. Our small sample size may have prevented us from detecting potentially subtle associations of early life OP exposure with these risk-taking behaviors.

**Supplementary Information:**

The online version contains supplementary material available at 10.1186/s12940-021-00822-y.

## Background

Prospective birth cohort studies have reported adverse neurodevelopment in association with prenatal organophosphate (OP) pesticide exposure [1–12]. In our Center for the Health Assessment of Mothers and Children of Salinas (CHAMACOS) study, a birth cohort set in a community living in proximity to agricultural fields in Salinas Valley, California, we have shown associations of prenatal urinary OP metabolites, a biomarker of exposure, with poorer neurodevelopment in childhood and early adolescence (through age 14 years), including poorer cognitive function [2], more hyperactive and inattentive behaviors [4], and poorer executive function [11] and social cognition [5].

Evidence suggests that poorer neurodevelopment in childhood may predict long-term problems, such as substance abuse, delinquency, violence, and other risk-taking behaviors in adolescence and early adulthood. For example, studies have observed that hyperactivity, impulsivity, and risk-taking behaviors at age 10 predict delinquency at ages 12–13 [13] and violence at ages 14 and 16 [14]. Children with attention deficit hyperactivity disorder (ADHD) are also more likely to engage in property theft, disorderly conduct, assault, and possession of a concealed weapon [15]; to be arrested [16], including for violent crimes [17, 18]; and to report alcohol, tobacco and illicit drug use [19, 20]. Furthermore, studies have reported that delinquent adolescents and criminal adult offenders have IQs averaging 6 to 11 points lower than those without a history of delinquency and criminality [21, 22].

Scientific literature suggests that early life exposure to lead, air pollution, and tobacco smoke may increase the risk of delinquent and criminal behavior in adolescence and early adulthood, even after controlling for socioeconomic status [23–35]. We recently reported mostly null associations of prenatal and childhood OP metabolite concentrations with juvenile delinquency up to age 16 years in CHAMACOS [36]. In the current study, we investigated associations of prenatal urinary OP metabolite concentrations with a broader group of risk-taking behaviors in CHAMACOS young adults up to age 18 years, including substance use, risky sexual activity, risky driving, and delinquency and police encounters.

## Methods

### Study sample

We recruited pregnant women from community clinics in California’s Salinas Valley between October 1999 and October 2000. These clinics primarily serve the Salinas Valley’s population of Mexican-origin agricultural workers and their families. Women were eligible to participate if they were ≥ 18 years old, spoke Spanish or English, qualified for low-income health insurance, were < 20 weeks gestation, and were planning to deliver at a local hospital. A total of 601 women were enrolled in the study, of whom 537 were followed through the birth of a live-born infant. Mother-child dyads were followed up every 1–2 years from 6 months to 18 years of age. A total of 317 young adults completed the protocol through age 18, of whom 315 (52% of the initial cohort) had mothers who had provided a urine sample prior to delivery; these participants comprised the study population included in the current analysis.

Written informed consent was obtained from mothers upon enrollment and follow up; children provided verbal assent starting at age 7, written assent starting at age 12, and full written consent at age 18. All activities were approved by the University of California, Berkeley Office for the Protection of Human Subjects.

### OP pesticide exposure assessment

Detailed descriptions of urine collection, analysis, and quality control procedures for dialkyl phosphates (DAPs), non-specific metabolites of OP exposure, have been presented elsewhere [37]. Briefly, urine samples were obtained from the mothers at approximately 13 weeks and 26 weeks gestation. Samples were aliquoted and stored at − 80 °C and then shipped on dry ice to the Centers for Disease Control and Prevention for analysis using gas chromatography-tandem mass spectrometry [38]. We quantified 6 dialkyl phosphate (DAP) metabolites, including three dimethylphosphate (DM) metabolites (dimethylphosphate, dimethyldithiophosphate, dimethylthiophosphate) and three diethylphosphate (DE) metabolites (diethylphosphate, diethyldithiophosphate, diethylthiophosphate). We summed their molar concentrations (nmol/L) to estimate total DAP metabolite concentrations. In CHAMACOS, DM metabolite concentrations greatly exceeded DE concentrations and were very similar to total DAP concentrations [37]. We therefore conducted analyses with only total DAPs.

We imputed metabolite values below the limit of detection using random imputation based on a log-normal probability distribution [37]. To correct for urine dilution, we corrected total DAP concentrations for specific gravity in all analyses [39]. We measured specific gravity using a refractometer (National Instrument Company, Inc., Baltimore, MD) calibrated with deionized water at room temperature.

### Assessment of risk-taking behaviors

At the 18-year study visit, adolescent participants completed a computer-based questionnaire that included questions about lifetime risk-taking behaviors. Adolescents were assured that their responses would be kept confidential to encourage candor in answering sensitive questions. We selected risk-taking behaviors across the following four domains: 1) substance use, 2) sexual activity, 3) driving, and 4) delinquency and police encounters.

#### Substance use

Questions on substance use were from the Monitoring the Future Survey (https://www.drugabuse.gov/drug-topics/trends-statistics/monitoring-future), and included items on alcohol, marijuana, and tobacco (smoked, vaped, or in other forms). From these data, we derived the following three dichotomous outcomes: 1) having been very drunk in the past 30 days; 2) smoking or vaping nicotine in the past 30 days; and 3) smoking or vaping marijuana in the past 30 days.

#### Sexual activity

Questions on sexual activity were from the Youth Risk Behavior Surveillance (YRBS) Survey [40], the National Institutes of Mental Health Risk Prevention Survey [41], and a Sexual Behavior Survey [42]. From these scales, we derived two dichotomous outcomes: 1) initiating sex before age 16 years; and 2) not always using a condom during vaginal sex.

#### Driving

Data on risky driving practices were drawn from the YRBS [40] and the Driver Behavior Rating Scale [43]. From these surveys we derived three dichotomous variables: 1) driving without a seat belt as the driver or passenger; 2) texting or looking at a mobile phone while driving; and 3) driving without a license.

#### Delinquency and police encounters

We created a checklist of delinquent or criminal behaviors which we adapted from the Self-Reported Delinquency and Self-Reported Behavior scales [44–46]. The checklist included items ranging from minor delinquency (e.g., lying about age to see a movie) to serious felony offences (e.g., arson, auto theft). Additional file 1 includes a full list of these delinquent acts. For each act, participants were asked to indicate whether they had ever committed the act, and if yes, their age when they first committed the act, and the total number of times they committed the act in their lifetime (1 time, 2–3 times, 4–5 times, or 6 or more times). Participants were also asked to report any instances of being “arrested, stopped, picked up, or questioned by the police” and to provide details on their age, the circumstances, and the outcome of each police encounter [45]. From this checklist, we derived two dichotomous outcomes: 1) committing at least one delinquent act; and 2) having an encounter with the police. We also examined delinquency and police encounters as counts, including: 1) number of different kinds of delinquent acts committed (i.e., a count of the unique types of acts in our delinquency checklist that the youth endorsed); 2) frequency of commission of delinquent acts (i.e., a sum of the number of times any delinquent acts were committed); and 3) number of police encounters. As an example, a youth who reported running away one time, shoplifting six or more times, and no other offenses would have committed two different kinds of delinquent acts with a total frequency of seven or more offenses.

### Assessment of adverse childhood experiences

We administered an adaptation of the Centers for Disease Control and Prevention (CDC) Adverse Childhood Experiences (ACE) survey [47] to young adult participants at the 18-year visit. The ACE survey includes questions about adverse events in the first 18 years of life and has shown good predictive validity [48]. Questions ranged from having enough to eat to physical and sexual abuse (see Additional file 2). Total scores ranged as low as 0 and were capped at 5.

### Covariate assessment

Study staff collected data on sociodemographic indicators at in-person visits conducted twice during pregnancy (13- and 26-weeks’ gestation), shortly following delivery, when children were 6 months, and every 1–2 years up to child’s age 18 years. At the child’s 6-month and 9-year visits, mothers were administered the Peabody Picture Vocabulary Test [49], or its Spanish equivalent [50], to assess their receptive language. Mothers were also administered the Center for Epidemiologic Studies Depression Scale (CES-D) at the child’s 1, 3, 7, and 9-year visits to assess their depressive symptoms [51]. Finally, mothers completed the Home Observation for the Measurement of the Environment (HOME)-Short Form at the child’s 6 months, 1, 2, 3.5, 7, 9, 10.5, and 12-year visits to assess enrichment in the home environment [52].

### Statistical analysis

We corrected DAP metabolite concentrations for specific gravity for each of the two pregnancy (13- and 26-week) urine samples and computed the mean value. We then log_10_ transformed mean DAP concentrations to reduce the influence of outliers and estimated associations with risk for outcomes across the four domains of risk-taking behaviors (substance use, sexual activity, driving, and delinquency/police encounters) per 10-fold increase in DAP concentrations using multivariable models. For dichotomous outcomes, we used generalized linear models with a log link and Poisson distribution. For count outcomes, we obtained incidence rate ratios using models with a negative binomial distribution to correct for overdispersion. In all cases we produced robust standard errors using the Huber-White sandwich estimator.

Based on previous studies examining of associations of DAPs with neurodevelopmental outcomes [2, 4, 5, 11], we selected the following covariates for inclusion in multivariable models: maternal age at delivery (continuous), education (≤6th grade, 7th–12th grade, high school graduate), years spent in the U.S. prior to delivery (≤5 years, > 5 years but non-native, native to U.S.), marital status at enrollment (married or living as married, not married or living as married), and depression (< 16, ≥16 on the CES-D, assessed at the 9-year visit); HOME z-score at the 6-month visit (continuous); and household poverty status at the time of assessment (at or below poverty level, > 100% of poverty level). We also included child sex and age at assessment (continuous), which are strongly related to outcomes, enhancing precision of estimates. For missing covariate values, we used the participant’s value from another proximal visit when possible and randomly imputed the small number of remaining missing values (*n* = 1 missing maternal depression, *n* = 1 missing HOME score).

We examined possible effect modification by two different factors: sex (males vs. females) and ACE score, dichotomized as low (0–2) vs. high (3+). In separate models, we included an interaction term between DAP concentrations and each modifier, derived stratum-specific effect estimates, and computed a Wald *p*-value for statistical interaction. Where we found associations of prenatal DAP concentrations with risk-taking behaviors, we used causal inference models [53] to assess mediation by neurodevelopmental outcomes for which we previously reported associations with DAPs, including IQ [2], attention [4, 8], and executive function [11].

## Results

As shown in Table 1, a large proportion of mothers in the CHAMACOS cohort (44.8%) had a ≤ 6th grade education at the time of delivery, almost half (47.6%) had lived in the U.S. ≤5 years prior to delivery, and most (83.2%) were married or living as married at that time. At the time of the 18-year visit, the majority of households (59.1%) were at or below the poverty line. Prenatal total DAP concentrations, averaged over the 13- and 26-week measures, were slightly higher among younger mothers, mothers not born in the U.S., and more well-educated and less impoverished mothers (Table 1). Participants included in the analysis (*n* = 315) differed only slightly from those not included (*n* = 222) as shown in Additional file 3; included participants had mothers who were slightly older and lived in the U.S. longer at the time of delivery and were more likely to be female.

**Table 1:** Sociodemographic and exposure characteristics of youth (*n* = 315) included in analysis: CHAMACOS study population, enrolled 1999–2000 in Salinas Valley, California

Covariate	n (%)	∑DAPs (nmol/L)
		GM ± GSD
Maternal age at delivery (years)		
18–24	128 (40.6)	145.8 ± 2.9
25–29	107 (34.0)	123.9 ± 2.8
30–34	51 (16.2)	131.6 ± 2.3
35–45	29 (9.2)	112.3 ± 2.8
Maternal education at baseline		
≤ 6th grade	141 (44.8)	127.1 ± 2.9
7th–12th grade	111 (35.2)	123.5 ± 2.6
High school grad or higher	63 (20.0)	164.2 ± 2.8
Years living in US prior to delivery		
≤ 5 years	150 (47.6)	134.8 ± 2.7
> 5 years, non-native	134 (42.5)	134.5 ± 2.8
Born in US	31 (9.8)	113.4 ± 2.8
Marital status at baseline		
Married or living as married	262 (83.2)	130.6 ± 2.8
Not married or living as married	53 (16.8)	141.7 ± 2.4
Maternal depression at 9-year visit (≥16 on CES-D)		
Yes	81 (25.7)	143.7 ± 2.5
No	234 (74.3)	128.8 ± 2.8
Young adult’s sex		
Male	143 (45.4%)	140.9 ± 2.7
Female	172 (54.6%)	125.8 ± 2.8
Household poverty at 18-year visit		
At or below poverty	129 (41.0)	119.0 ± 2.8
> 100% poverty level	186 (59.1)	142.6 ± 2.7
	GM (GSD)	
HOME z-score at 6 months	0.02 (1.06)	
Young adult’s age at 18-year visit	18.2 (0.3)	
Total DAP concentrations (nmol/L)	132.4 ± 2.7	
Total DAP concentrations, specific gravity adjusted	171.5 ± 2.8	

The prevalence of risk-taking behaviors ranged from 8.9% (smoking or vaping nicotine) to 70.2% (committing any delinquent act) (Table 2). Risk-taking behaviors, including substance use and risky sex, were most common among youth born to non-U.S. born mothers who were living in the U.S. > 5 years at the time of their child’s birth (Additional file 4). Substance use, delinquent acts, and police encounters were considerably more common among males than females (Additional file 4).

**Table 2:** Adjusted^a^ relative risk (RR) of risk-taking behaviors reported at 18 years per 10-fold increase in mean prenatal total urinary DAP concentrations (nmol/gL), overall and stratified by sex, in the CHAMACOS study population, enrolled 1999–2000 in Salinas Valley, California

Outcome	Total N	N (%) with outcome	RR (95% CI)^a^	Males RR (95% CI)^a^	Females RR (95% CI)^a^	*p*-value for interaction
Substance Use (Y/N)^b^						
Very drunk in last 30 days	315	30 (9.5)	0.96 (0.43, 2.15)	1.53 (0.55, 4.25)	0.65 (0.20, 2.16)	0.27
Smoked/vaped nicotine in last 30 days	315	28 (8.9)	1.89 (1.00, 3.56)	1.46 (0.56, 3.79)	2.50 (1.10, 5.66)	0.41
Smoked/vaped marijuana in the past last 30 days	315	78 (24.8)	1.11 (0.71, 1.73)	1.26 (0.72, 2.19)	0.99 (0.52, 1.89)	0.56
Sex (Y/N)^b^						
Sex before age 16	311	47 (15.1)	1.16 (0.64, 2.12)	1.91 (0.84, 4.34)	0.81 (0.34, 1.92)	0.15
Doesn’t always use a condom	310	70 (22.6)	0.98 (0.63, 1.51)	0.91 (0.46, 1.82)	1.02 (0.59, 1.78)	0.79
Driving (Y/N)^b^						
Doesn’t always use seatbelt (driver or passenger)	315	102 (32.4)	1.03 (0.72, 1.45)	1.19 (0.63, 2.23)	0.94 (0.62, 1.43)	0.55
Texts/looks at phone while driving	221	110 (49.8)	0.85 (0.63, 1.15)	0.81 (0.51, 1.27)	0.88 (0.59, 1.31)	0.77
Drives without license (regularly)	221	96 (43.4)	1.74 (1.25, 2.42)	2.18 (1.35, 3.50)	1.46 (0.93, 2.28)	0.23
Delinquency/police encounters (Y/N)^b^						
Police encounters	315	77 (24.4)	1.16 (0.76, 1.78)	1.25 (0.70, 2.24)	1.07 (0.57, 2.02)	0.72
Any delinquent act	315	221 (70.2)	0.97 (0.83, 1.13)	1.09 (0.90, 1.32)	0.88 (0.69, 1.11)	0.15
Delinquency/police encounters (counts)^c^		median (25–75%ile)	IRR (95% CI)^a^	Males IRR (95% CI)^a^	Females IRR (95% CI)^a^	
Police encounters	315	0 (0–0)	1.56 (0.91, 2.70)	2.10 (0.97, 4.54)	1.10 (0.51, 2.38)	0.24
Delinquent acts: number of different types of acts	315	2 (0–6)	1.01 (0.76, 1.35)	1.29 (0.88, 1.88)	0.85 (0.56, 1.27)	0.13
Delinquent acts: frequency of behaviors	315	8 (0–34)	1.13 (0.75, 1.69)	1.51 (0.86, 2.64)	0.90 (0.52, 1.55)	0.19

^a^ Adjusted for maternal age at delivery (continuous), years spent in the US prior to delivery (≤5 years, > 5 years but non-native, native to US), education (≤6th grade, 7th–12th grade, high school graduate), marital status at enrollment (married or living as married vs not married or living as married), and depression (< 16, ≥16 on the CES-D, assessed at the 9-year visit); HOME z-score at the 6-month visit (continuous variable); household poverty status at the time of assessment (at or below poverty level, > 100% of poverty level); and young adult’s sex and age at assessment (continuous variable)

^b^ Log risk (Poisson) models

^c^ Negative binomial models

As shown in Table 2, associations of total prenatal DAPs with risk-taking behaviors were generally null and imprecise, with two exceptions: a 10-fold increase in total prenatal DAP concentrations was associated with a higher risk of smoking or vaping nicotine within the past 30 days (relative risk [RR] = 1.89, 95% CI: 1.00, 3.56) and driving without a license (RR = 1.74, 95% CI: 1.25, 2.42).

Associations across sex were inconsistent, mostly hovering at the null (Table 2). There was slightly higher risk for smoking or vaping nicotine within the past 30 days with a 10-fold increase in total prenatal DAP concentrations among females (RR = 2.50, 95% CI: 1.10, 5.66) vs. males (RR = 1.46, 95% CI: 0.56, 3.79; *p*-value for interaction = 0.41). In addition, there was a slightly higher risk for driving without a license with a 10-fold increase in total prenatal DAP concentrations among males (RR = 2.18, 95% CI: 1.35, 3.50) vs. females (RR = 1.46, 95% CI: 0.93, 2.28; *p*-value for interaction = 0.23). Finally, there was a pattern of slightly higher risk associated with prenatal DAP concentrations for delinquent acts and police encounters among males (e.g., for counts of police encounters IRR_males_ = 2.10, 95% CI: 0.97,4.54 vs. IRR _females_ = 1.10, 95% CI: 0.51, 2.38; *p*-value for interaction = 0.24). None of these sex differences were statistically significant and stratum specific estimates were imprecise.

We also found little difference in DAP-risk taking behaviors across low and high ACE scores (Table 3). There was slightly higher risk for smoking/vaping nicotine in the past 30 days in association with DAP concentrations for those with high ACE scores (RR = 2.49, 95% CI: 0.95, 6.54) vs. those with low ACE scores (RR = 1.41, 95% CI: 0.55, 3.60), but confidence intervals were wide and the difference was not statistically significant (*p*-value for interaction = 0.43). Similar differences were found for counts of police encounters, but were again imprecise (IRR_highACEs_ = 2.53, 95% CI: 1.07, 6.02 vs. IRR_lowACEs_ = 1.39, 95% CI: 0.69, 2.83; *p*-value for interaction = 0.30). The only statistically significant difference associated with prenatal DAP concentrations was for counts of delinquency acts, though in the opposite direction than expected (IRR_highACEs_ = 0.72, 95% CI: 0.36, 1.45 vs. IRR_lowACEs_ = 1.76, 95% CI: 1.06, 2.92; *p*-value for interaction = 0.04).

**Table 3:** Adjusted^a^ relative risk (RR) of risk-taking behaviors reported at 18 years per 10-fold increase in mean prenatal total urinary DAP concentrations (nmol/gL) stratified by ACEs, subsetting on participants with ACEs (*n* = 309) and dichotomized as low ACEs (< 3; *n* = 220) and high ACEs (3+; *n* = 89), in the CHAMACOS study population, enrolled 1999–2000 in Salinas Valley, California

Outcome	Total N	N (%) with outcome	Low ACEs RR (95% CI)^a^	High ACEs RR (95% CI)^a^	*p*-value for interaction
Substance Use (Y/N)^b^					
Very drunk in last 30 days	308	28 (9.1)	0.63 (0.20, 4.04)	1.11 (0.36, 3.40)	0.50
Smoked/vaped nicotine in last 30 days	309	26 (8.4)	1.41 (0.55, 3.60)	2.49 (0.95, 6.54)	0.43
Smoked/vaped marijuana in the past last 30 days	309	75 (24.3)	1.16 (0.65, 2.07)	0.96 (0.51, 1.79)	0.65
Sex (Y/N)^b^					
Sex before age 16	305	47 (15.4)	1.52 (0.78, 2.94)	0.81 (0.26, 2.56)	0.35
Doesn’t always use a condom	305	69 (22.6)	1.00 (0.55, 1.80)	0.87 (0.43, 1.77)	0.77
Driving (Y/N)^b^					
Doesn’t always use seatbelt (driver or passenger)	309	98 (31.7)	0.87 (0.55, 1.37)	1.15 (0.62, 2.15)	0.49
Texts/looks at phone while driving	215	106 (49.3)	0.81 (0.55, 1.18)	0.88 (0.54, 1.42)	0.78
Drives without license (regularly)	215	91 (42.3)	1.74 (1.09, 2.75)	1.61 (0.98, 2.65)	0.83
Delinquency/police encounters (Y/N)^b^					
Police encounters	309	75 (24.3)	1.10 (0.59, 2.06)	1.22 (0.69, 2.15)	0.81
Any delinquent act	309	217 (70.2)	1.01 (0.83, 1.24)	0.87 (0.67, 1.12)	0.34
Delinquency/police encounters (counts)^c^		median (25–75%ile)	Low ACEs IRR (95% CI)^a^	High ACEs IRR (95% CI)^a^	
Police encounters	309	0 (0–0)	1.39 (0.69, 2.83)	2.53 (1.07, 6.02)	0.30
Delinquent acts: number of different types of acts	309	2 (0–6)	1.10 (0.77, 1.56)	0.98 (0.62, 1.56)	0.71
Delinquent acts: frequency of behaviors	309	8 (0–34)	1.76 (1.06, 2.92)	0.72 (0.36, 1.45)	0.04

^a^ Adjusted for maternal age at delivery (continuous), years spent in the US prior to delivery (≤5 years, > 5 years but non-native, native to US), education (≤6th grade, 7th–12th grade, high school graduate), marital status at enrollment (married or living as married vs not married or living as married), and depression (< 16, ≥16 on the CES-D, assessed at the 9-year visit); HOME z-score at the 6-month visit (continuous variable); household poverty status at the time of assessment (at or below poverty level, > 100% of poverty level); and young adult’s sex and age at assessment (continuous variable)

^b^ Log risk (Poisson) models

^c^ Negative binomial models

Largely null associations of DAP concentrations with risk-taking behaviors precluded us from examining mediation by neurodevelopmental outcomes.

## Discussion

We did not find any strong or consistent associations of prenatal OP pesticide exposure with risk-taking behaviors, including substance use, risky sexual activity, risky driving, and delinquency/police encounters among 18-year-old young adults in CHAMACOS. While there were some isolated associations of DAP concentrations with higher risk for smoking/vaping and for driving without a license, the absence of any consistent patterns made these findings less credible. In addition, there were no consistent differences in these associations across potential modifiers, including sex and childhood adversity. The mostly null associations may be due to the number of CHAMACOS study participants with measured prenatal DAP concentrations and 18-year follow-up data (*n* = 315), which may be too small to detect potentially subtle associations of prenatal OP pesticide exposure with risk-taking behaviors.

Previous studies have demonstrated that early life exposure to environmental toxicants can have life-long sequelae, in line with the early origins of disease theory [54, 55]. For example, early life exposure to lead has been shown to be linked to adverse consequences in adolescence and early adulthood, such as delinquency, substance abuse, and criminality [23–32]. Previous studies have not examined the downstream consequences of early life pesticide exposure, however. Much of the previous epidemiologic literature on OP pesticides has focused on neurodevelopmental outcomes in childhood. We recently reported that prenatal and childhood OP exposure was associated with mostly null associations with juvenile delinquency at age 16 years in CHAMACOS [36]. In the current study, we investigated how these associations manifest more broadly in risk-taking behavior as these children transition to adulthood.

We hypothesized that one of the primary pathways for associations of prenatal OP exposure with risk-taking behaviors is through associations with neurodevelopment. Previous studies, including CHAMACOS, report associations of prenatal DAPs with poorer cognition [2, 6, 7], as well as behaviors related to ADHD [4, 11, 56]. Suspected mechanisms underlying associations of low-level OPs (not high enough to inhibit acetylcholinesterase) with neurodevelopment include inhibition of axonal growth [57, 58], increased oxidative stress [59–61], and epigenetic modifications [62, 63]. However, as essentially all associations of DAP concentrations with risk-taking behaviors were null, examination of mediation by neurodevelopment was moot.

California accounts for about 25% of all U.S. agricultural pesticide use and in 2012, 9.2 million pounds of pesticides were applied in Monterey County, where Salinas is located [64]. Higher OP pesticide exposure has been shown in CHAMACOS, with women’s urinary DAP concentrations averaging 40% higher, though within range, of those in the representative NHANES sample of U.S. women of child-bearing age [37]. These high exposures, coupled with adverse sociodemographic conditions in Salinas, including early life adversity, creates conditions that may place youth at risk for increased risk-taking behavior in adolescence/early adulthood. In the current study we did not find clear or consistent differences by pesticide exposure measures across childhood adversity (high vs. low ACEs) in CHAMACOS (Table 3).

Overall, low study power was the largest limitation of this study, especially in models stratified by sex or adversity. Many of the outcomes we assessed in relation to risk-taking behavior are serious outcomes, including substance use, unprotected sex, and delinquent behavior. The importance of investigating these associations cannot be overstated, given the pervasiveness of pesticide exposures and the serious nature of these outcomes. Further study of these associations in larger samples should be undertaken.

Another limitation of this study concerns exposure assessment. DAPs are non-specific metabolites and cannot be attributed to any single OP pesticide [65]. In addition, OPs are rapidly metabolized in the body [66], making exposure measurement error a central concern. We were able to offset this limitation somewhat with two pregnancy measures. And while non-differential measurement error could have explained the null associations found in this study, we have reported associations of maternal DAP metabolite concentrations with several developmental outcomes in CHAMACOS [2, 4, 5, 8, 11].

We did not investigate associations of postnatal OP exposure with high-risk behavior. While there was certainly postnatal OP exposure in this community living in proximity to agriculture, associations of neurodevelopment with early childhood DAP exposure has been shown to be weaker that prenatal exposure in CHAMACOS and other cohorts, as previously reported [2, 4, 12]. We therefore focused on prenatal exposure for the current analysis. However, given that neurodevelopment continues into early adulthood, it will be important to investigate associations with postnatal exposure throughout childhood and adolescence with these outcomes in future studies.

There were also notable strengths of this study. CHAMACOS is a prospective cohort recruited during pregnancy with high exposure to pesticides and adversity. We were able to take repeated measures of exposure during pregnancy, a critical window of exposure for brain development and therefore a potentially important window of exposure with respect to high-risk behavior. We also frequently followed up CHAMACOS participants with detailed outcome assessment including information about risk taking behaviors in youth.

## Conclusions

In summary, we did not observe associations between prenatal OP exposure and risk-taking behaviors in adolescence/early adulthood in the CHAMACOS population. Larger studies may be needed to examine the potentially subtle associations of OPs with these risk-taking behaviors. This research is especially important given the gravity of these outcomes and the high prevalence of pesticide exposure in the population.

## Supplementary Information

**Additional file 1.**

**Additional file 2.**

**Additional file 3.**

**Additional file 4.**

## Data Availability

The datasets generated during and/or analyzed during the current study are not publicly available due to protection of personal identifiers and the sensitive nature of these data, but are available from the corresponding author on reasonable request.

## Abbreviations

ADHD: Attention deficit hyperactivity disorder
CES-D: Center for Epidemiologic Studies Depression Scale
CHAMACOS: Center for the Health Assessment of Mothers and Children of Salinas
CI: Confidence interval
DAP: Dialkyl phosphate
DE: Diethyl phosphate
DM: Dimethyl phosphate
HOME: Home Observation for Measurement of the Environment
OP: Organophosphate
RR: Relative risk
YRBS: Youth Risk Behavior Surveillance
