# Supplementary material for: Prenatal exposure to organophosphate pesticides and risk-taking behaviors in early adulthood
Source: Environ Health. 2022 Jan 10;21:8. doi: 10.1186/s12940-021-00822-y (PMC8751255; doi:10.1186/s12940-021-00822-y)
Supplement: Supplementary file 1 — Additional file 1. [file 12940_2021_822_MOESM1_ESM.docx]

Additional File 1. Types of delinquent acts inventoried at 18 years. Items derived from the Self-Reported Delinquency Scale [1] and the Self-Reported Behavior Scale [2].

| Have you ever… | |
| --- | --- |
| Run away from home? | Taken something from a car that did not belong to you? |
| Run away from home and stayed overnight? | Knowingly bought, sold, or held stolen goods, or tried to do any of these things? |
| Lied about your age to get into some place or to buy something? | Gone joyriding, that is, taken a motor vehicle, such as a car or motorcycle, for a ride or drive without the owner’s permission? |
| Trespassed? | Stolen or tried to steal a motor vehicle such as a car or motorcycle? |
| Made obscene telephone calls, such as calling someone and saying dirty things? | Used checks illegally, or used a slug or fake money to pay for something (including intentional overdrafts)? |
| Made a false 911 call? | Used or tried to use credit cards or bank cards without the owner’s permission? |
| Hitchhiked where it was illegal to do so? | Tried to cheat someone by selling them something that was worthless or not what you said it was? |
| Carried a hidden weapon? | Verbally threatened or intimidated someone? |
| Been loud, rowdy, or unruly in a public place so that people complained about it or got you in trouble? | Hit (or threatened to hit) a teacher or other adult at school? |
| Begged for money or things from strangers? | Hit (or threatened to hit) your parent(s)? |
| Been drunk in a public place? | Hit (or threatened to hit) other students? |
| Purposely damaged or destroyed property belonging to your parent(s) or other family members? | Hit (or threatened to hit) anybody else? |
| Purposely damaged or destroyed property belonging to a school? | Gotten into a fight at school or work? |
| Purposely damaged or destroyed other property that did not belong to you? | Gotten into a fight in your neighborhood? |
| Purposely set fire to a house, building, car or other property, or tried to do so? | Hit someone with the idea of seriously hurting them? |
| Avoided paying for things such as movie, bus or subway rides, food, or computer services? | Attacked someone with a weapon or with the idea of seriously hurting them? |
| Gone into or tried to go into a building to steal something? | Used a weapon, force or strong-arm methods to get money or things from people? |
| Stolen or tried to steal things worth $5 or less? | Thrown rocks or bottles at people? |
| Stolen or tried to steal things worth between $5 and $50? | Been involved in a gang fight? |
| Stolen or tried to steal things worth between $50 and $100? | Sold marijuana or hashish? (weed, pot, grass, hash) |
| Stolen or tried to steal something worth $100 or more? | Sold hard drugs such as heroin, cocaine (coke), meth (crystal, shard), LSD (acid), or ecstasy (molly, MDMA)? |
| Stolen money or other things from your parent(s) or other members of your family? | Been paid for having sexual relations with someone? |
| Stolen money or other things from anyone else (other than members of your family)? | Physically hurt or threatened to hurt someone to get them to have sex with you? |
| Taken something from a store without paying for it? | Had or tried to have sexual relations with someone against their will (other than those you just mentioned)? |
| Snatched someone’s purse or wallet or picked someone’s pocket? |  |

**REFERENCES**

1. Hinshaw SP, Owens EB, Sami N, Fargeon S: **Prospective follow-up of girls with attention-deficit/hyperactivity disorder into adolescence: Evidence for continuing cross-domain impairment**. *Journal of consulting and clinical psychology* 2006, **74**(3):489-499.

2. Van Hulle CA, Rodgers JL, D'Onofrio BM, Waldman ID, Lahey BB: **Sex differences in the causes of self-reported adolescent delinquency**. *Journal of abnormal psychology* 2007, **116**(2):236-248.
