# Supplementary material for: Prenatal exposure to organophosphate pesticides and risk-taking behaviors in early adulthood
Source: Environ Health. 2022 Jan 10;21:8. doi: 10.1186/s12940-021-00822-y (PMC8751255; doi:10.1186/s12940-021-00822-y)
Supplement: Supplementary file 2 — Additional file 2. [file 12940_2021_822_MOESM2_ESM.docx]

Additional File 2. Adapted ACES

1. Many children experience stressful life events while they are growing up, during their first 18 years of life. Please read the **seven** statements below. Count the number of statements that apply to you.

| **While you were growing up, during your first 18 years of life:**   - A parent or other adult in the household **often** swore at you, insulted you, put you down, humiliated you, or acted in a way that made you afraid that you might be physically hurt. - A parent or other adult in the household **often** pushed, grabbed, slapped, or threw something at you or **ever** hit you so hard that you had marks or were injured. - An adult or person at least 5 years older than you **ever** touched or fondled you, had you touch their body in a sexual way, or attempted or actually had oral, anal, or vaginal intercourse with you. - Your parents were **ever** separated or divorced. - You lived with someone who was a problem drinker or alcoholic or who used street drugs. - ​A household member was depressed, mentally ill, or attempted suicide. - A household member went to prison. |
| --- |

How many of these statements apply to you?

- 0
- 1
- 2
- 3
- 4
- 5 or more

While you were growing up, during your first 18 years of life:

2A. Did you **often** feel that no one in your family loved you or thought you were important or special?

- No
- Yes [SKIP TO 3A]

2B. Did you **often** feel that your family didn’t look out for each other, feel close to each other, or support each other?

- No
- Yes

3A. Did you **often** feel that you didn’t have enough to eat, had to wear dirty clothes, and had no one to protect you?

- No
- Yes [SKIP TO 4A]

3B. Did you **often** feel that your parents were too drunk or high to take care of you or take you to the doctor if you needed it?

- No
- Yes

4A. Was your mother or stepmother **often** pushed, grabbed, slapped, or had something thrown at her?

- No
- Yes [SKIP TO END]

4B. Was your mother or stepmother **sometimes or often** kicked, bitten, hit with a fist, or hit with something hard?

- No
- Yes [SKIP TO END]

4C. Was your mother or stepmother **ever** repeatedly hit at least a few minutes or threatened with a gun or knife?

- No
- Yes

**END**
