# Supplementary material for: Prenatal exposure to organophosphate pesticides and risk-taking behaviors in early adulthood
Source: Environ Health. 2022 Jan 10;21:8. doi: 10.1186/s12940-021-00822-y (PMC8751255; doi:10.1186/s12940-021-00822-y)
Supplement: Supplementary file 3 — Additional file 3. [file 12940_2021_822_MOESM3_ESM.docx]

Additional File 3. Baseline characteristics for CHAMACOS cohort included in analysis (n=315) compared with those not included in analysis (n=222).

|  | Included in analysis  n (%) | Not included in analysis  n (%) |
| --- | --- | --- |
| Covariate |  |  |
| Total | 315 (100.0) | 222 (100.0) |
|  |  |  |
| Maternal age at delivery (years) |  |  |
| 18-24 | 128 (40.8) | 122 (55.0) |
| 25-29 | 107 (34.0) | 57 (25.7) |
| 30-34 | 51 (16.2) | 32 (14.4) |
| 35-45 | 29 (9.2) | 11 (5.0) |
|  |  |  |
| Maternal education at baseline |  |  |
| ≤6th grade | 141 (44.8) | 93 (41.9) |
| 7th-12th grade | 111 (35.2) | 83 (37.4) |
| High school grad or higher | 63 (20.0) | 46 (20.7) |
|  |  |  |
| Years living in US prior to delivery |  |  |
| ≤5 years | 150 (47.6) | 124 (55.9) |
| >5 years, non-native | 134 (42.5) | 69 (31.1) |
| Born in US | 31 (9.8) | 29 (13.1) |
|  |  |  |
| Marital status at baseline |  |  |
| Married or living as married | 262 (83.2) | 170 (76.6) |
| Not married or living as married | 53 (16.8) | 52 (23.4) |
|  |  |  |
| Young adult sex |  |  |
| Male | 143 (45.4%) | 123 (55.4) |
| Female | 172 (54.6%) | 99 (44.6) |
|  |  |  |
| Household poverty at baseline |  |  |
| At or below poverty | 192 (61.0) | 140 (63.1) |
| >100% poverty | 123 (39.1) | 82 (36.9) |
