# Supplementary material for: Prenatal exposure to organophosphate pesticides and risk-taking behaviors in early adulthood
Source: Environ Health. 2022 Jan 10;21:8. doi: 10.1186/s12940-021-00822-y (PMC8751255; doi:10.1186/s12940-021-00822-y)
Supplement: Supplementary file 4 — Additional file 4. [file 12940_2021_822_MOESM4_ESM.docx]

Additional File 4. Percent with risk-taking behaviors by sociodemographic characteristics of youth (n=315) in the CHAMACOS study population, enrolled 1999-2000 in Salinas Valley, California.

|  |  | Substance use in past 30 days (%) | | | | |  | | Risky sex (%) | | |  | | Risky driving (%) | | | | |  | | Delinquency/police encounters (%) | | | | |
| --- | --- | --- | --- | --- | --- | --- | --- | --- | --- | --- | --- | --- | --- | --- | --- | --- | --- | --- | --- | --- | --- | --- | --- | --- | --- |
| Covariate | n | very drunk | | | smoked/vaped nicotine | smoked/vaped marijuana |  | | had sex <16y | | doesn't always wear a condom |  | | doesn't always use seatbelt | texts while driving | | drives without license | |  | | police encounter  <18y | | committed any delinquent act | |  |
| Maternal age at delivery |  |  | | |  |  |  | |  | |  |  | |  |  | |  | |  | |  | |  | |  |
| 18-24 | 128 | 10.9 | | | 12.5 | 26.7 |  | | 16.5 | | 20.5 |  | | 28.9 | 51.8 | | 44.6 | |  | | 30.5 | | 70.3 | |  |
| 25-29 | 107 | 5.6 | | | 4.7 | 18.7 |  | | 13.3 | | 21.0 |  | | 31.8 | 50.0 | | 45.0 | |  | | 20.6 | | 69.2 | |  |
| 30-34 | 51 | 9.8 | | | 5.9 | 19.6 |  | | 15.7 | | 24.0 |  | | 29.2 | 52.6 | | 34.2 | |  | | 21.6 | | 70.6 | |  |
| 35-45 | 29 | 12.7 | | | 13.8 | 34.5 |  | | 14.3 | | 35.7 |  | | 27.9 | 35.0 | | 50.0 | |  | | 17.2 | | 72.4 | |  |
| *Pearson 𝜒2 p-value* |  | *0.24* | | | *0.12* | *0.11* |  | | *0.92* | | *0.35* |  | | *0.53* | *0.57* | | *0.62* | |  | | *0.22* | | *0.99* | |  |
|  |  |  | | |  |  |  | |  | |  |  | |  |  | |  | |  | |  | |  | |  |
| Maternal education at baseline | | | | |  |  |  | |  | |  |  | |  |  | |  | |  | |  | |  | |  |
| ≤6th grade | 141 | 8.5 | | | 11.4 | 22.7 |  | | 17.3 | | 12.2 |  | | 33.3 | 51.0 | | 42.9 | |  | | 22.0 | | 72.3 | |  |
| 7th-12th grade | 111 | 10.8 | | | 6.3 | 26.1 |  | | 11.0 | | 17.4 |  | | 31.5 | 42.1 | | 47.4 | |  | | 27.0 | | 64.9 | |  |
| High school grad or higher | 63 | 9.5 | | | 7.9 | 27.0 |  | | 17.5 | | 30.1 |  | | 31.8 | 59.6 | | 38.3 | |  | | 25.4 | | 74.6 | |  |
| *Pearson 𝜒2 p-value* |  | *0.82* | | | *0.36* | *0.74* |  | | *0.33* | | *0.15* |  | | *0.95* | *0.16* | | *0.61* | |  | | *0.64* | | *0.30* | |  |
|  |  |  | | |  |  |  | |  | |  |  | |  |  | |  | |  | |  | |  | |  |
| Years living in US prior to delivery | | | | | |  |  | |  | |  |  | |  |  | |  | |  | |  | |  | |  |
| ≤5 years | 150 | 6.7 | | | 7.3 | 18.7 |  | | 14.9 | | 16.3 |  | | 29.3 | 41.6 | | 44.6 | |  | | 21.3 | | 65.3 | |  |
| >5 years, non-native | 134 | 11.9 | | | 10.5 | 32.8 |  | | 15.9 | | 30.3 |  | | 35.8 | 57.7 | | 43.3 | |  | | 26.1 | | 75.4 | |  |
| Born in US | 31 | 12.9 | | | 9.7 | 19.4 |  | | 12.9 | | 19.4 |  | | 32.3 | 52.2 | | 39.1 | |  | | 32.3 | | 71.0 | |  |
| *Pearson 𝜒2 p-value* |  | *0.25* | | | *0.65* | ***0.02*** |  | | *0.91* | | ***0.02*** |  | | *0.51* | *0.07* | | *0.89* | |  | | *0.37* | | *0.18* | |  |
|  |  |  | | |  |  |  | |  | |  |  | |  |  | |  | |  | |  | |  | |  |
| Marital status at baseline |  |  | | |  |  |  | |  | |  |  | |  |  | |  | |  | |  | |  | |  |
| Married or living as married | 262 | 9.5 | | | 8.8 | 25.6 |  | | 14.3 | | 22.6 |  | | 26.4 | 48.9 | | 44.5 | |  | | 21.4 | | 69.9 | |  |
| Not married | 53 | 9.4 | | | 9.4 | 20.8 |  | | 18.9 | | 22.6 |  | | 33.6 | 53.9 | | 38.5 | |  | | 39.6 | | 71.7 | |  |
| *Pearson 𝜒2 p-value* |  | *0.98* | | | *0.88* | *0.46* |  | | *0.40* | | *0.99* |  | | *0.31* | *0.58* | | *0.49* | |  | | ***0.01*** | | *0.79* | |  |
|  |  |  | | |  |  |  | |  | |  |  | |  |  | |  | |  | |  | |  | |  |
| Maternal depression at 9-year visit (≥16 on CES-D) | | | | | | | |  | |  |  | |  |  | |  | |  | |  | |  | |  | |
| Depressed | 81 | | 11.1 | | 11.1 | 24.7 |  | | 11.5 | | 21.8 |  | | 25.9 | 45.0 | | 45.0 | |  | | 25.9 | | 65.4 | |  |
| Not depressed | 234 | | 9.0 | | 8.1 | 24.8 |  | | 16.3 | | 22.8 |  | | 34.6 | 51.6 | | 42.9 | |  | | 23.9 | | 71.8 | |  |
| *Pearson 𝜒2 p-value* |  | | *0.57* | | *0.42* | *0.99* |  | | *0.31* | | *0.85* |  | | *0.15* | *0.39* | | *0.78* | |  | | *0.72* | | *0.28* | |  |
|  |  | |  | |  |  |  | |  | |  |  | |  |  | |  | |  | |  | |  | |  |
| Young adult’s sex |  | |  | |  |  |  | |  | |  |  | |  |  | |  | |  | |  | |  | |  |
| Male | 143 | | 10.5 | | 11.9 | 30.1 |  | | 16.3 | | 21.3 |  | | 30.1 | 51.9 | | 47.2 | |  | | 32.2 | | 79.0 | |  |
| Female | 172 | | 8.7 | | 6.4 | 20.4 |  | | 14.1 | | 23.7 |  | | 34.3 | 47.8 | | 39.8 | |  | | 18.0 | | 62.8 | |  |
| *Pearson 𝜒2 p-value* |  | | *0.59* | | *0.09* | ***0.05*** |  | | *0.59* | | *0.62* |  | | *0.42* | *0.55* | | *0.27* | |  | | ***<0.01*** | | ***<0.01*** | |  |
|  |  | |  | |  |  |  | |  | |  |  | |  |  | |  | |  | |  | |  | |  |
| Household poverty at 18-year visit | | | |  |  |  |  | |  | |  |  | |  |  | |  | |  | |  | |  | |  |
| At or below poverty | 129 | | 10.9 | | 8.5 | 26.4 |  | | 16.5 | | 21.3 |  | | 30.2 | 44.0 | | 46.2 | |  | | 29.5 | | 70.5 | |  |
| >100% poverty | 186 | | 8.6 | | 9.1 | 23.7 |  | | 14.1 | | 23.5 |  | | 33.9 | 52.9 | | 41.5 | |  | | 21.0 | | 69.9 | |  |
| *Pearson 𝜒2 p-value* |  | | *0.50* | | *0.85* | *0.59* |  | | *0.56* | | *0.64* |  | | *0.50* | *0.15* | | *0.50* | |  | | *0.09* | | *0.90* | |  |
